# The regulatory toll-like receptor 4 genetic polymorphism rs11536889 is associated with renal, coagulation and hepatic organ failure in sepsis patients

**DOI:** 10.1186/1479-5876-12-177

**Published:** 2014-06-21

**Authors:** Ashham Mansur, Luisa von Gruben, Aron F Popov, Maximilian Steinau, Ingo Bergmann, Daniel Ross, Michael Ghadimi, Tim Beissbarth, Martin Bauer, José Hinz

**Affiliations:** 1 Department of Anesthesiology, University Medical Center, Georg August University, D-37075 Goettingen, Germany; 2 Department of Cardiothoracic Transplantation & Mechanical Support, Royal Brompton and Harefield Hospital, Harefield, Hill End Road, UB9 6JH London, UK; 3 Department of General and Visceral Surgery, University Medical Center, Georg August University, D-37075 Goettingen, Germany; 4 Department of Medical Statistics, University Medical Center, Georg August University, D-37075 Goettingen, Germany

**Keywords:** Toll-like receptor 4, Single-nucleotide polymorphism (SNP), Intensive care unit, Organ failure marker, SOFA scores

## Abstract

**Background:**

Toll-like receptor 4 (TLR4), a lipopolysaccharide (LPS) receptor complex signal-transducing molecule, plays a crucial role in sensing LPS from gram-negative bacteria. TLR4 signaling pathway activation by LPS plays a major role in sepsis pathogenesis. A single nucleotide polymorphism, rs11536889, in the 3’-untranslated region of the *TLR4* gene is thought to affect TLR4 translation. This study aimed to investigate whether organ failure in sepsis patients is related to the *TLR4* rs11536889 genotype.

**Methods:**

Adult Caucasian patients with sepsis from the intensive care unit of a university medical center were followed up for 90 days, and organ failure was recorded as the primary outcome variable. Blood samples were collected at enrollment for *TLR4* rs11536889 genotyping. Sepsis-related organ failure assessment (SOFA) scores were quantified at sepsis onset and throughout the observational period to monitor organ failure.

**Results:**

A total of 210 critically ill patients with sepsis were enrolled into this study. Wild-type GG was compared to GC/CC. During their stay in the intensive care unit, GG patients presented significantly higher SOFA scores than did C allele carriers (7.9 ± 4.5 and 6.8 ± 4.2, respectively; p = 0.0005). Analysis of organ-specific SOFA sub-scores revealed significant differences in three organ systems: renal, coagulation and hepatic (p = 0.0005, p = 0.0245 and p < 0.0001, respectively). Additionally, the rs11536889 polymorphism was associated with a higher incidence of gram-negative infections.

**Conclusions:**

These results offer the first evidence that *TLR4* rs11536889 is a useful marker of organ failure in patients with sepsis.

## Background

Sepsis is a major health challenge. Despite improved treatment options, sepsis remains a leading cause of death in intensive care units [1]. Lipopolysaccharide (LPS), or endotoxin, the major outer membrane component of gram-negative bacteria, is a potent inflammatory response stimulator [2]. In addition, LPS triggers inflammation in gram-negative sepsis [3]. Excessive amounts of gut-derived LPS released during intestinal hypo-perfusion have also been implicated in sepsis caused by gram-positive and fungal infections [4,5]. LPS signaling is initiated by the activation of the myeloid differentiation factor 2 and toll-like receptor 4 (TLR4) complexes on myeloid cells [2,6]. TLR4 has recently been shown to recognize endogenous danger-type, or ‘alarmin,’ factors, thereby implicating TLR4 as a tissue injury and microbial invasion sensor [7]. Studies using mouse strains deficient in TLR4 signaling [8,9] or expression [10-13] or those using TLR inhibitors in wild-type mice [14,15] confirmed that TLR4 contributes to bacterial clearance and the host inflammatory response in the infection setting [16].

Two missense single nucleotide polymorphisms (SNPs) in the *TLR4* gene, Asp299Gly/Thr399Ile, have been reported to be associated with endotoxin hypo-responsiveness to inhaled LPS [17]. This investigation was followed by a series of studies that explored the potential impact of these SNPs on the incidence and course of infectious diseases [18], such as septic shock with gram-negative bacterial infection [19]. Although some studies have shown a relevance of the Asp299Gly/Thr399Ile SNPs in gram-negative infections, others did not confirm this association [20-22]. Furthermore, recent studies using primary cells isolated from individuals bearing these mutations have indicated that the Asp299Gly/Thr399Ile haplotype has little or no effect on LPS responsiveness [23].

Recently, Sato et al. demonstrated the biological significance of a genetic variation of the *TLR4* gene called rs11536889. Functional analyses revealed that *TLR4* rs11536889 contributes to the translational regulation of TLR4 expression and has some influence on the response to LPS, possibly by binding to microRNAs, which act in post-transcriptional regulation [24]. A large study that included prostate cancer patients and age-matched controls from Sweden revealed an association between *TLR4* rs11536889 and prostate cancer [25]. Later, Hishida et al. observed that *TLR4* rs11536889 genotypes are associated with severe gastric atrophy in *helicobacter pylori*-seropositive Japanese subjects [26]. Zhou et al. found that the *TLR4* rs11536889 SNP is significantly associated with *hepatitis type B* virus recurrence after liver transplantation [27]. In addition, Miedema et al. found that this SNP is associated with an increased risk of chemotherapy-induced neutropenia in children with acute lymphoblastic leukemia [28]. These observations suggest that the rs11536889 genetic variation of the TLR4 gene may influence human inflammatory and/or malignant diseases [24].

This study aimed at exploring whether the putative regulatory *TLR4* rs11536889 genotypes relate to organ failure severity in critically ill patients with sepsis during their time in the intensive care unit. The outcomes of wild-type GG were compared to those of GC/CC.

## Methods

### Patients

Adult Caucasian patients admitted to the University Medical Center Goettingen (UMG) intensive care units (ICUs) between April 2012 and May 2013 were screened daily according to the American College of Chest Physicians/Society of Critical Care Medicine (ACCP/SCCM) criteria for sepsis, severe sepsis, or septic shock [29,30]. Caucasian origin was assessed by questioning the patients, their next of kin or their legal representatives. The patient exclusion criteria were as follows: 1. age younger than 18 years; 2. pregnancy, nursing an infant, or planning to become pregnant or nurse an infant; 3. receiving an immunosuppressive therapy (e.g., cyclosporine or azathioprine) or cancer-related chemotherapy; 4. a documented or suspected acute myocardial infarction within the previous six weeks; 5. a history of New York Heart Association functional class IV chronic heart failure: 6. human immunodeficiency virus infection or end-stage process (e.g., progressive multifocal leukoencephalopathy or systemic *Mycobacterium avium* infection); 7. morbidity and death were considered imminent, the patient was classified as “do not resuscitate” or “do not treat”, or the patient and/or a legally authorized representative was not committed to aggressive management; 8. the patient was not expected to survive the observation period of 28 days or was not likely to be given life support because of a preexisting, uncorrectable medical condition, including a poorly controlled neoplasm, end-stage lung disease, or home oxygen requirement; 9. the patient was in a chronic vegetative state or had a similar long-term neurologic condition; 10. participation in any other investigational study (drug or device); 11. the patient was unwilling or unable to be fully evaluated during the study period; and 12. the patient was a study-site employee or was an immediate family member of a study-site employee involved in the study. The study was approved by the University of Goettingen ethics committee, Goettingen, Germany (15/1/12) and conformed to the Declaration of Helsinki ethical principles (Seoul, 2008). Written informed consent was obtained either from patients or their legal representatives.

### Data collection

The Sequential Organ Failure Assessment (SOFA) [31] and Acute Physiology and Chronic Health Evaluation (APACHE) II [32] scores were evaluated at the onset of sepsis. Organ function was assessed subsequently on days 2, 3, 5, 7, 14, 21 and 28, and organ failure was quantified, with the SOFA score as the primary outcome variable. Patients were followed up for a maximum of 90 days, and their deaths were recorded as a secondary outcome variable. The necessity of mechanical ventilation, vasopressor administration, or renal-replacement therapy as well as the ICU duration was recorded as secondary variables.

### TLR4 rs11536889 genotyping

Peripheral blood monocytes (PBMCs) from approximately 30 ml of heparinized peripheral blood were isolated through Ficoll density gradient centrifugation according to standard procedures described previously [33]. Cell preparations were routinely assessed for viability (>95%) by trypan blue dye exclusion.

Genomic DNA (gDNA) was purified from PBMCs using the AllPrep DNA Mini Kit (Qiagen, Hilden, Germany) according to the manufacturer’s protocol. The isolated nucleic acid concentration and purity were determined by 260 and 280 nm optical density readings. DNA integrity was evaluated through 0.6% agarose gel electrophoresis.

Genotyping was performed using 4 ng of PBMC-derived gDNA in a commercially available genotyping assay (Assay ID C_31784034_10, Applied Biosystems, Darmstadt, Germany) in a total volume of 10 μl. The reactions were performed in a StepOnePlus sequence detection system (Applied Biosystems, Darmstadt, Germany) according to the supplier’s instructions.

### Statistical analyses

The Hardy-Weinberg equilibrium exact test for deviation was performed using an online calculator, which was provided by the Institute of Human Genetics, Helmholtz Center Munich, Germany (http://ihg.gsf.de/cgi-bin/hw/hwa1.pl). Statistical analyses were performed with the Statistica (StatSoft, Tulsa, Oklahoma, USA, version 10) or R (The R Foundation for Statistical Computing, version 3.0.0) software. Significance, based on contingency tables, was calculated using two-sided Fisher’s exact or chi-square tests, as appropriate. Two continuous variables were compared using the Mann-Whitney test. To estimate the significance of the clinical observations over the 28-day period, we fitted a linear regression model with the parameters day, genotype, and genotype-day interaction. The results were visualized by calculating the means and 95% confidence intervals (CIs) from normal distributions at each time point. Time-to-event data were compared using the log-rank test from the Statistica package survival. A p-value less than 0.05 was considered significant.

## Results

### Study population

A total of 212 adult Caucasian patients with sepsis were enrolled into this study. Two patients were excluded; one patient fulfilled an exclusion criterion, as a B cell lymphoma diagnosis became known, and the other patient was excluded because informed consent was withdrawn by his legally authorized representative. Subsequently, the study population comprised 210 patients, in which 134 were male (64%), and 76 were female (36%; Table 1). The patient ages ranged from 19 to 91 (median, 65). The sepsis subtypes were sepsis/severe sepsis (n = 100) and septic shock (n = 110). At baseline, the patient disease severity SOFA and APACHE II scores were 8.6 ± 4.1 and 21.3 ± 7.4, respectively (Table 1). The comorbidities comprised hypertension, myocardial infarction history, chronic obstructive pulmonary disease (COPD), renal dysfunction, diabetes mellitus, chronic liver diseases, cancer history, and stroke history (Table 1).

**Table 1:** **Patient baseline characteristics with regard to the *TLR4 *** **rs11536889 genotypes**

	**All n = 210**	**GG n = 146**	**GC/CC n = 64**	***p*** **value**
Age [years]	63 ± 15	63 ± 16	63 ± 15	0.9116
Male, %	64%	46%	58%	0.2752
Body-mass index, mean ± SD	28 ± 9	28 ± 7	30 ± 13	0.1507
Severity of sepsis	48%	46%	52%	0.4576
Sepsis/severe sepsis, %	52%	54%	48%	
Septic shock, %				
SOFA score, mean ± SD	8.6 ± 4.1	8.9 ± 4.3	8.0 ± 3.7	0.2449
APACHE II score, mean ± SD	21.3 ± 7.4	21.6 ± 7.4	20.6 ± 7.6	0.4053
Comorbidities, %				
Hypertension		58	59	1.0000
History of myocardial infarction		5	6	0.7586
COPD		18	17	1.0000
Renal dysfunction		11	20	0.0834
Diabetes mellitus (NIDDM)		11	8	0.6202
Diabetes mellitus (IDDM)		11	5	0.1938
Chronic liver diseases		7	9	0.7839
History of cancer		18	19	0.8481
History of stroke		6	8	0.7647
Recent surgical history, %				0.0631
Elective surgery	28	31	19	
Emergency surgery	44	44	44	
No history of surgery	28	24	37	
Site of infection, %				0.1516
Lung	50	46	56	
Abdomen	30	31	28	
Bone or soft tissue	7	7	6	
Surgical wound	2	3	0	
Urogenital	1	0	3	
Primary bacteremia	6	7	3	
Other	4	5	3	
Organ support, %				
Mechanical ventilation	83	83	83	0.8933
Use of vasopressor	52	54	48	0.4576
Renal-replacement therapy	10	11	9	0.7300

The data are presented as the mean ± SD or percentages.

### Disease severity at sepsis onset

*TLR4* rs11536889 was successfully genotyped in all subjects. The genotype distribution was 146:62:2 (GG:GC:CC), which was consistent with the Hardy-Weinberg equilibrium (p = 0.12). The resulting 0.16 minor allele frequency was similar to that given for Caucasians in public databases. The rs11536889 GC and CC genotypes were pooled together because the size of the CC genotype group was too small (n = 2). Subsequently, patients of genotype GG were compared to that of CG/CC. There was no difference regarding age, gender, or body mass index related to the *TLR4* rs11536889 genotype. A comparison between the G homozygous patients and C allele carriers revealed no significant difference between the proportion of patients with sepsis/severe sepsis and septic shock at baseline (day 1 of sepsis; p = 0.4576). Furthermore, there were no SOFA and APACHE II score differences regarding the *TLR4* rs11536889 genotypes at sepsis onset, and there were no significant preexisting conditions differences between the *TLR4* rs11536889 genotypes (Table 1). Moreover, the recent surgical histories and primary infection sites showed no significant difference with respect to the genotype distribution (Table 1).

### Disease progression and mortality

**Figure 1:**
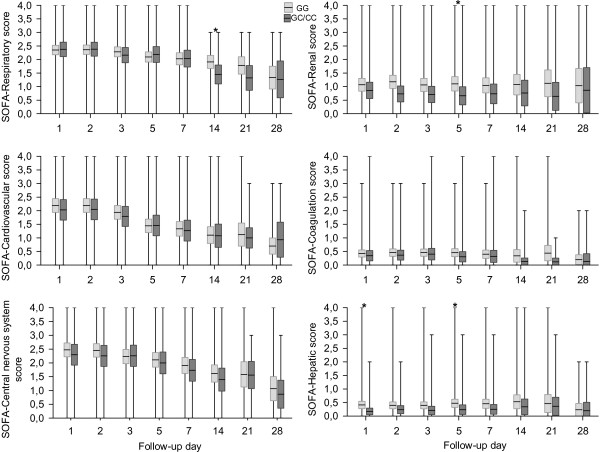
**SOFA sub-scores by genotype on each day during the follow-up period.** The means are indicated by horizontal bars. The boxes are limited by the 25th and 75th percentile. The whiskers represent the minimum and maximum. The differences were not significant except where indicated by*.

**Figure 2:**
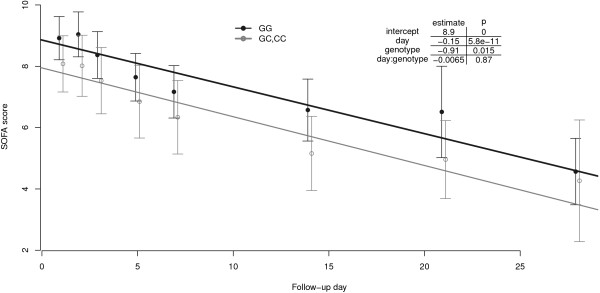
**SOFA score by genotype during the follow-up period.** The means and 95% normal CIs plus regression lines are displayed. The box displays the coefficients and P values from a linear regression model that models the SOFA scores as a function of the follow-up day and genotype.

Disease progression was monitored by SOFA score changes during the patient ICU stays. The scores and the need for organ support were recorded on days 1, 2, 3, 5, 7, 14, 21, and 28. Although no differences in disease scores were observed at sepsis onset, the TLR4 rs11536889 GG patients experienced significantly higher SOFA scores over time (p = 0.0005) compared with the C allele carriers (Table 2). The three organ-specific SOFA scores were significantly different between the two groups; the GG patients presented higher SOFA-renal scores (p = 0.0005), SOFA-coagulation scores (p = 0.0245), and SOFA-hepatic scores (p < 0.0001; Table 2, Figure 1). An overall linear model was fitted to the values, which included the various time points. This model also revealed a significant genotype effect; the GG patients presented higher SOFA scores than did the GC/CC subjects (p = 0.015; Figure 2). The mean SOFA scores of the two CC patients were 11.6 ± 3.5 (mean ± SD). Among all SOFA scores, the minimum and maximum were 0 and 23, respectively.

**Table 2:** **Disease progression with regard to the *TLR4 *** **rs11536889 genotypes**

	**All n = 210**	**GG n = 146**	**GC/CC n = 64**	***p *** **value**
SOFA	7.6 ± 4.5	7.9 ± 4.5	6.8 ± 4.2	0.0005
SOFA-Respiratory score	2.1 ± 1.1	2.1 ± 1.1	2.0 ± 1.1	0.1950
SOFA-Cardiovascular score	1.6 ± 1.5	1.7 ± 1.5	1.4 ± 1.4	0.1296
SOFA-Central nervous system score	2.0 ± 1.5	2.1 ± 1.5	1.9 ± 1.4	0.0802
SOFA-Renal score	0.9 ± 1.4	1.0 ± 1.4	0.7 ± 1.2	0.0005
SOFA-Coagulation score	0.3 ± 0.8	0.4 ± 0.8	0.3 ± 0.7	0.0245
SOFA-Hepatic score	0.3 ± 0.7	0.4 ± 0.8	0.2 ± 0.5	<0.0001
Mortality analysis, %:				
Death at day 28	18	18	17	1.0000
Death at day 90	30	30	28	0.8698
Length of stay in ICU (days)	18 ± 16	18 ± 17	20 ± 14	0.0720
Organ support^*^, %				
Mechanical ventilation		75	74	0.6286
Use of vasopressor		38	37	0.8426
Renal replacement therapy		14	9	0.4486

The data are presented as the mean ± SD or percentages.

^*^Based on the total number of observations in follow-up days.

The 28-day and 90-day mortality analyses yielded no significant result between the groups (p = 1.0000 and p = 0.8698, respectively; Table 2 (Additional file 1: Figure S1)). Both at the beginning and over the observational period, there was no significant difference between the GG patients and C allele carriers regarding organ support requirement (mechanical ventilation, vasopressor use, and renal replacement therapy; Tables 1 and 2). The mean ICU stay duration of the GG survivors did not differ significantly from that of the GC/CC survivors (Table 2). Additionally, there was a significantly higher gram-negative infection incidence rate among the C allele carriers (81%) compared with that in the GG patients (62%; p = 0.0062) (Table 3).

**Table 3:** Infection types over the observational period

	**GG n = 146**	**GC/CC n = 64**	***p *** **value**
Infection type			
Gram-negative	62%	81%	0.0062
Gram-positive	83%	83%	1.0000
Fungal	52%	58%	0.4565
Viral	18%	17%	1.0000
Parasitic	0%	0%	
Other	1%	3%	

## Discussion

The present study addressed the question of whether the putative regulatory *TLR4* rs11536889 genotypes are related to organ failure in critically ill patients with sepsis.

The primary endpoint, organ failure, was quantified using SOFA scores as a specific clinical marker in patients with sepsis and was significantly higher in *TLR4* rs11536889 GG patients compared with those of *TLR4* rs11536889 GC and CC patients (Table 2).

The *TLR4* rs11536889 genotype distribution among the septic patients was similar to database entries regarding healthy Caucasians and also followed the Hardy-Weinberg equilibrium. The *TLR4* rs11536889 genotypes, however, were not associated with any of the recorded baseline characteristics. As assessed by scoring the sepsis type (sepsis and severe sepsis vs. septic shock) and SOFA and APACHE II scores, we found that the *TLR4* rs11536889 genotypes were also not related to the septic disease severity at sepsis onset (Table 1). We believe that the SOFA scores at sepsis onset did not differ between the GG versus GC and CC genotypes because of the phenotypic heterogeneity of the sepsis syndrome. This heterogeneity is influenced by many factors, including the pathogenic organism responsible for the infection, its location, and the amount of time passed since the onset of infection, as well as other individual parameters. To detect genotypic differences, a longitudinal observation involving SOFA scores quantified over the study period is much more promising (Table 2).

As shown by Sato et al., monocytes from *TLR4* rs11536889 CC subjects expressed significantly higher levels of TLR4 compared with those from TLR4 rs11536889 GG and GC subjects. When PBMCs were stimulated with LPS, a TLR4 ligand, the cells from the *TLR4* rs11536889 CC and GC subjects secreted significantly higher levels of the proinflammatory cytokine IL-8 compared to cells from the GG subjects [24]. Accordingly, these previous investigations support the assumption that *TLR4* rs11536889 GG sepsis patients present severe organ dysfunction (as measured using SOFA scores) because of attenuated TLR4 proinflammatory signaling in response to LPS compared to C allele carriers.

These significant results, with respect to organ dysfunction, reveal severe morbidity among *TLR4* rs11536889 GG patients (according to SOFA scores) and together with the fact that GG patients are assumed to present attenuated TLR4 expression [24], offer an explanation why synthetic TLR4 antagonists have failed to produce a clinical benefit in patients with severe sepsis [14,15]. These agents may alter the inflammatory response via TLR4 to pathogens, thereby contributing to organ dysfunction in these patients.

The SOFA-renal score was higher in *TLR4* rs11536889 GG patients, indicating severe renal dysfunction in this group. Because *TLR4* rs11536889 GG patients may exhibit decreased *TLR4* expression, our results are consistent with former observations indicating that decreased TLR4 expression in chronic kidney disease patients was associated with attenuated proinflammatory cytokine synthesis during infection [34]. The observed severe hepatic dysfunction measured using the SOFA-hepatic score among *TLR4* rs11536889 GG subjects, indicating severe hyperbilirubinemia in this group, is in agreement with recent findings reported by Deng et al. [16] that TLR4 signaling is essential for LPS clearance by hepatocytes during sepsis. The absence of an association between the rs11536889 genotypes and the SOFA respiratory score may be attributed to the fact that the SOFA respiratory score is somewhat weak because it only refers to the oxygenation index. This score depends on several factors, such as ventilator settings during mechanical ventilation and different ventilator settings that result in different oxygenation indices, which lead to score variation. We believe that there was no significant difference in the SOFA-CNS score between the genotypes mainly because sepsis patients are treated with sedating medication, which impacts the CNS and thus affects the SOFA-CNS score. The SOFA-Cardiovascular score most likely did not differ between GG patients and C allele carriers because this score is only based on the catecholamine therapy needed, which depends on volumetric status and cardiac function.

Analysis of the 28-day and 90-day mortality revealed no significant differences among the *TLR4* rs11536889 genotypes. The severe organ failure observed in G homozygous patients may not have contributed to higher mortality rates because the patients received sufficient intensive care treatment, which allowed their organ failures to be managed appropriately. The patients were treated according to current guidelines for the treatment of sepsis (Surviving Sepsis Campaign) [35].

Additionally, there was a significantly higher gram-negative infection rate among the C allele carriers (81%) compared with the rate observed in the GG patients (62%; p = 0.0062) (Table 3). This result is in agreement with previous observations linking this polymorphism with an increased susceptibility to infection [24,26,27]. This observation of higher susceptibility of C allele carriers to gram-negative infections should be thoroughly examined in future studies to detect any causality between the SNP and susceptibility to gram-negative infections.

A possible limitation of this study is the possibility that the studied *TLR4* rs11536889 SNP associated with organ failure in patients with sepsis is in linkage disequilibrium with SNPs in another nearby gene and that these latter genes are responsible for the observed phenotypic effects.

To the best of our knowledge, this is the first investigation evaluating this putative regulatory polymorphism in this key innate immune receptor in adult Caucasian sepsis patients, showing a significant association between the *TLR4* rs11536889 GG genotype and the severity of organ failure (renal, coagulation and hepatic). According to these results, it would be worthwhile to further assess the *TLR4* rs11536889 polymorphism for its relevance to sepsis in larger and independent cohorts.

## Conclusions

This study assesses the validity of the assumption that a well-known regulatory *TLR4* polymorphism influences the outcome of sepsis among adults. An analysis of organ-specific SOFA sub-scores revealed significant differences in three organ systems: renal, coagulation and hepatic. These results offer the first evidence that *TLR4* rs11536889 is a useful marker of organ failure in patients with sepsis. This polymorphism should be assessed for its organ failure relevance in sepsis in larger, independent cohorts.

## Abbreviations

APACHE: Acute physiology and chronic health evaluation; CNS: Central nervous system; COPD: Chronic obstructive pulmonary disease; ICU: Intensive care unit; LPS: Lipopolysaccharide; PBMCs: Peripheral blood monocytes; SOFA: Sepsis-related organ failure assessment; TLR4: Toll-like receptor 4.

## Competing interests

The authors declare no competing interests.

## Authors’ contributions

All authors have contributed to the study design, the acquisition of data (clinical and experimental), or the analysis and interpretation of data. Specifically, LVG and MS primarily performed sample and clinical data collection and TLR4 laboratory analyses. MS, AP, IB, DR, MG and MB participated in study design and supervised patient enrollment and clinical data monitoring. TB contributed to the study design and conception, performed the bioinformatics and performed and approved statistical analyses. AM and JH designed the study, supervised sample and data collection, performed analyses and drafted the manuscript. All authors were involved either in manuscript drafting or its revision. All authors have approved the final version of the manuscript.

## Supplementary Material

Additional file 1 **Kaplan-Meier survival analysis.** The Kaplan-Meier curve shows the survival curves censored at day 90 for the TLR4 rs11536889 GG and GC/CC genotypes. A mortality risk among the patients under study did not differ between the two groups. Click here for file
