# Supplementary figures and images for: The regulatory toll-like receptor 4 genetic polymorphism rs11536889 is associated with renal, coagulation and hepatic organ failure in sepsis patients
Source: J Transl Med. 2014 Jun 21;12:177. doi: 10.1186/1479-5876-12-177 (PMC4085654; doi:10.1186/1479-5876-12-177)

Kaplan-Meier survival analysis

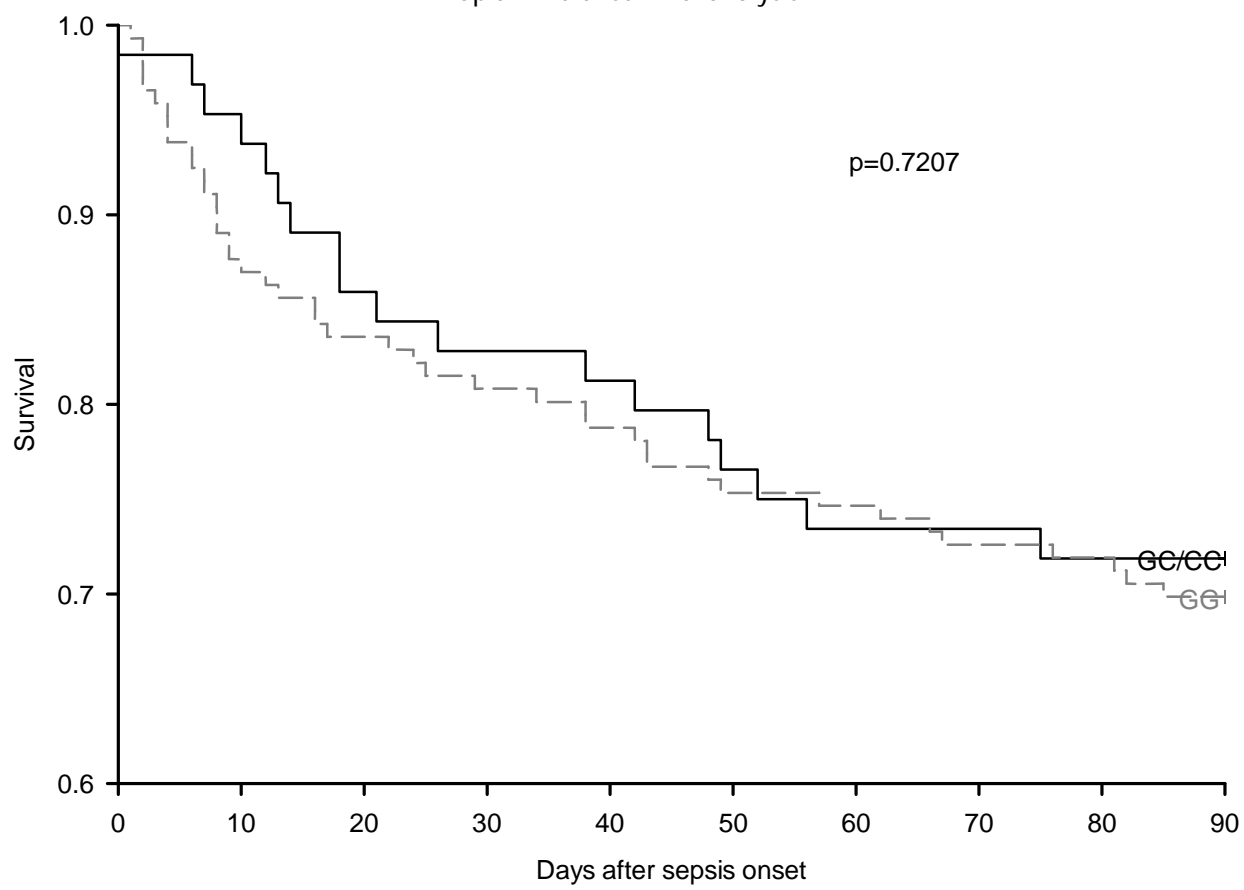

Supplement: Additional file 1 — Kaplan-Meier survival analysis. The Kaplan-Meier curve shows the survival curves censored at day 90 for the TLR4 rs11536889 GG and GC/CC genotypes. A mortality risk among the patients under study did not differ between the two groups. [file 1479-5876-12-177-S1.pdf]
